# Comparison the accuracy of a novel implant robot surgery and dynamic navigation system in dental implant surgery: an *in vitro* pilot study

**DOI:** 10.1186/s12903-023-02873-8

**Published:** 2023-03-28

**Authors:** Jianping Chen, Xiaolei Bai, Yude Ding, Liheng Shen, Xin Sun, Ruijue Cao, Fan Yang, Linhong Wang

**Affiliations:** 1 Center for Plastic & Reconstructive Surgery, Department of Stomatology, Zhejiang Provincial People’s Hospital, Affiliated People’s Hospital, Hangzhou Medical College, Hangzhou, Zhejiang China; 2 Department of Stomatology, Zhejiang Provincial People’s Hospital, Affiliated People’s Hospital, Hangzhou Medical College, Hangzhou, Zhejiang China; 3 Department of Stomatology, Zhejiang Chinese Medical University, Hangzhou, Zhejiang China

**Keywords:** Dental implant robotic system, Dynamic navigation system, Dental implant, Accuracy

## Abstract

**Background:**

To compare the accuracy of dental implant placement using a novel dental implant robotic system (THETA) and a dynamic navigation system (Yizhimei) by a *vitro* model experiment.

**Methods:**

10 partially edentulous jaws models were included in this study, and 20 sites were randomly assigned into two groups: the dental implant robotic system (THETA) group and a dynamic navigation system (Yizhimei) group. 20 implants were placed in the defects according to each manufacturer’s protocol respectively. The implant platform, apex and angle deviations were measured by fusion of the preoperative design and the actual postoperative cone-beam computed tomography (CBCT) using 3D Slicer software. Data were analyzed by *t* - test and Mann-Whitney *U* test, *p* < 0.05 was considered statistically significant.

**Results:**

A total of 20 implants were placed in 10 phantoms. The comparison deviation of implant platform, apex and angulation in THETA group were 0.58 ± 0.31 mm, 0.69 ± 0.28 mm, and 1.08 ± 0.66^°^ respectively, while in Yizhimei group, the comparison deviation of implant platform, apex and angulation were 0.73 ± 0.20 mm, 0.86 ± 0.33 mm, and 2.32 ± 0.71^°^ respectively. The angulation deviation in THETA group was significantly smaller than the Yizhimei group, and there was no significant difference in the deviation of the platform and apex of the implants placed using THETA and Yizhimei, respectively.

**Conclusion:**

The implant positioning accuracy of the robotic system, especially the angular deviation was superior to that of the dynamic navigation system, suggesting that the THETA robotic system could be a promising tool in dental implant surgery in the future. Further clinical studies are needed to evaluate the current results.

## Background

The accuracy of implant placement including its position, angle, and depth within the jawbone is essential and would affect its long-term stability, survival and success rate [1]. The traditional freehand operation depends on the clinical experience of the doctor and the accuracy of the operation. Any small error and deviation can affect the three-dimensional position of the implant and its long-term efficacy, or even destroy important anatomical structures and cause serious complications [2]. As a result, static navigation systems, dynamic navigation systems and robotic systems have emerged to assist in implant surgery, thus improving surgical precision, achieving long-term stability of the implant, reducing surgical complications and improving patient comfort. Static surgical templates guide the surgery to improve the accuracy of implantation site and orientation, but there are still some limitations, such as the choice of implant guide support, the presence of barriers to cooling, a limited field of view, the inability to dynamically adjust the design, and the thickness of the guide affecting the surgical operating space [3, 4]. In recent years, digital medicine has been proposed and rapidly developed, enabling faster and more accurate visualization of surgical procedures. Digital medicine-based preoperative planning, surgical templates and video navigation have been widely used for implant placement [5].

Computer-assisted dynamic navigation allows tracking the position of the drilling needle during the implant procedure, avoiding damage to adjacent important anatomical tissue and preventing surgical complications [6]. It is worth noting that the dynamic navigation system improves the accuracy of the implant position, depth and angle, and significantly reduces the operation time [7]. Yizhimei is an active infrared navigation system for surgery. Through the virtual visualization of medical images, infrared light positioning technology can locate surgical instruments and patients in real time, achieve accurate real-time navigation of surgery, and effectively improve the accuracy and efficiency of implant surgery. Robotic-assisted surgical systems have been a hot research topic in the last decade due to the good stability of the robotic arm and its ability to reduce the labor intensity of the operator. Yomi is the first reported surgical robot and it is a fully assisted dental implant surgical robot developed by Neocis, Inc. received approval from the U.S. Food and Drug Administration (FDA) in 2017 [8, 9]. In the same year, Zhao Yimin’s team developed the first autonomous dental implant surgery robot, which enables interpretation and reproduction of the anatomical structure of the surgical area, accurate pre-implantation design, automatic and precise intraoperative positioning, real-time surgical navigation and calibration [10].

The novel THETA robotic dental implant system, developed by Hangzhou Jianjia Robot Co. LTD, is a semi-automatic system, which could conduct positioning, drilling and implant placement according to control the integrated button (line setting button, teaching button) with an optical navigation system. All wrist joints of UR-3e manipulator can rotate 360 degrees, and the end joints can rotate infinitely. With force sensors, UR-3e manipulator can cooperate well with users in the same space through force position coupling control and handle high-precision tasks.

At present, there are CBCT image errors, registration errors, positioning and marking device printing errors, and visual system errors in both dynamic navigation system and robotic dental implant system. Few studies comparing the accuracy of the two implant systems. The accuracy of the implant-assisted technique is evaluated mainly through model experiments and clinical trials. In this study, a phantom experimental design in vitro was selected to compare the accuracy of a dental implant robotic system with a dynamic navigation system.

## Materials and methods

### The experimental operational procedure

**Fig. 1:**
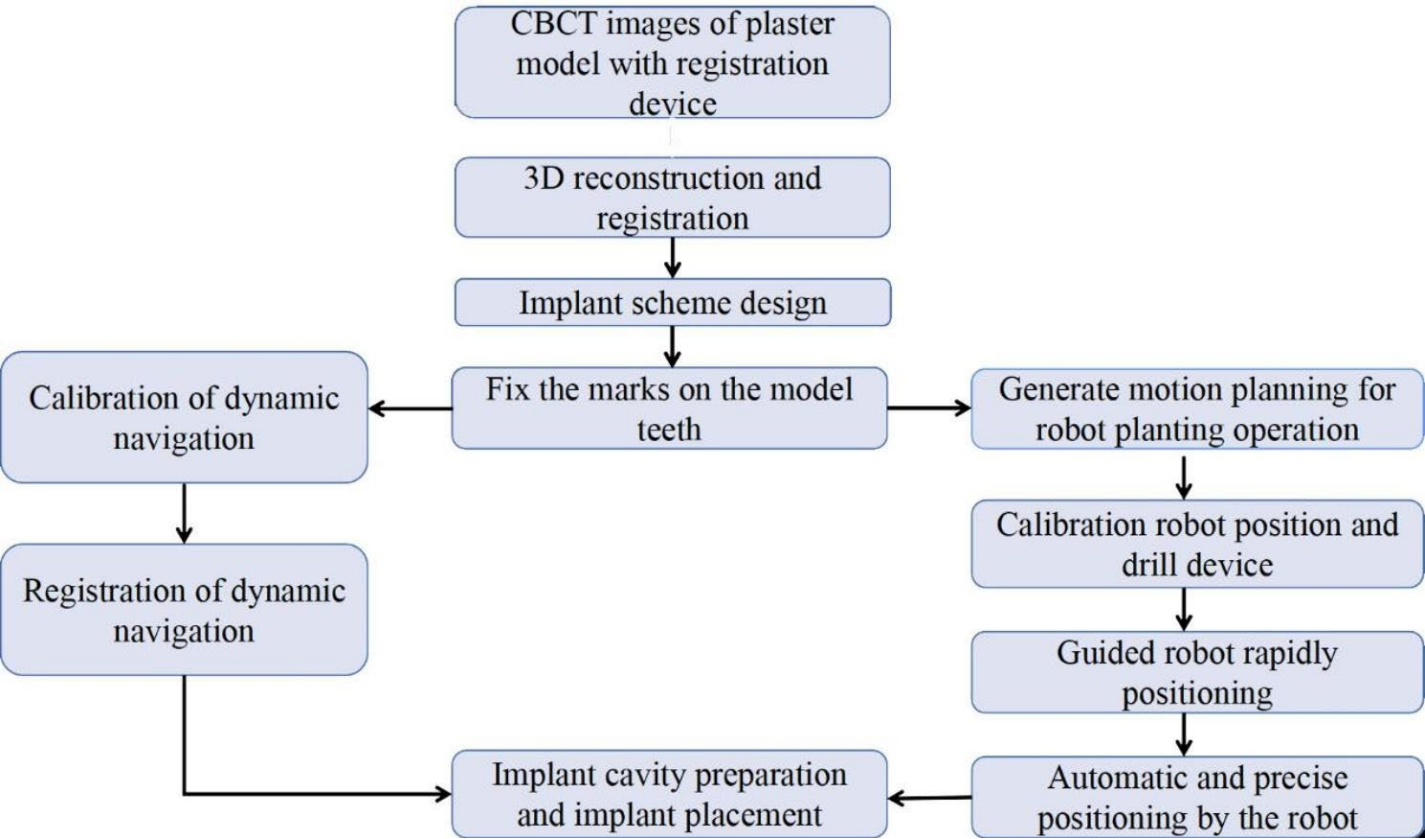
Experimental operation procedure with robotic system and dynamic navigation system

10 partially edentulous models containing 20 tooth missing sites were included in this study, and randomly divided into two groups, the tooth position, bone density and implant information were shown in Table 1, and the experimental operational procedure was shown in Fig. 1. The sample size was calculated from the results of the previous pre-experiment. All clinical procedures were performed by the same clinician. The operator practiced before the formal experiment, and the sample size was planned according to the results of the preliminary experiment.

**Table 1:** Detail of experimental groups

Group	Serial number	Bone density	Tooth Sites	Implant
THETA	A1	II	45 43	Nobel PCC RP 4.3 × 11.5 mm Nobel PCC NP 3.5 × 8 mm
Yizhimei	A2	II	45 43	Nobel PCC RP 4.3 × 11.5 mm Nobel PCC NP 3.5 × 8 mm
THETA	B1	II	12 21	Nobel PCC NP 3.5 × 8 mm Nobel PCC RP 4.3 × 10 mm
Yizhimei	B2	II	12 21	Nobel PCC NP 3.5 × 8 mm Nobel PCC RP 4.3 × 10 mm
THETA	C1	III	45 46	Nobel PCC RP 4.3 × 10 mm Nobel PCC RP 4.3 × 11.5 mm
Yizhimei	C2	III	45 46	Nobel PCC RP 4.3 × 10 mm Nobel PCC RP 4.3 × 11.5 mm
THETA	D1	II	36 37	Nobel PCC RP 4.3 × 10 mm Nobel PCC RP 4.3 × 11.5 mm
Yizhimei	D2	II	36 37	Nobel PCC RP 4.3 × 10 mm Nobel PCC RP 4.3 × 11.5 mm
THETA	E1	III	14 16	Nobel PCC RP 4.3 × 10 mm Nobel PCC RP 5.0 × 10 mm
Yizhimei	E2	III	14 16	Nobel PCC RP 4.3 × 10 mm Nobel PCC RP 5.0 × 10 mm

### THETA robotic-assisted dental implant surgery procedure

**Fig. 2:**
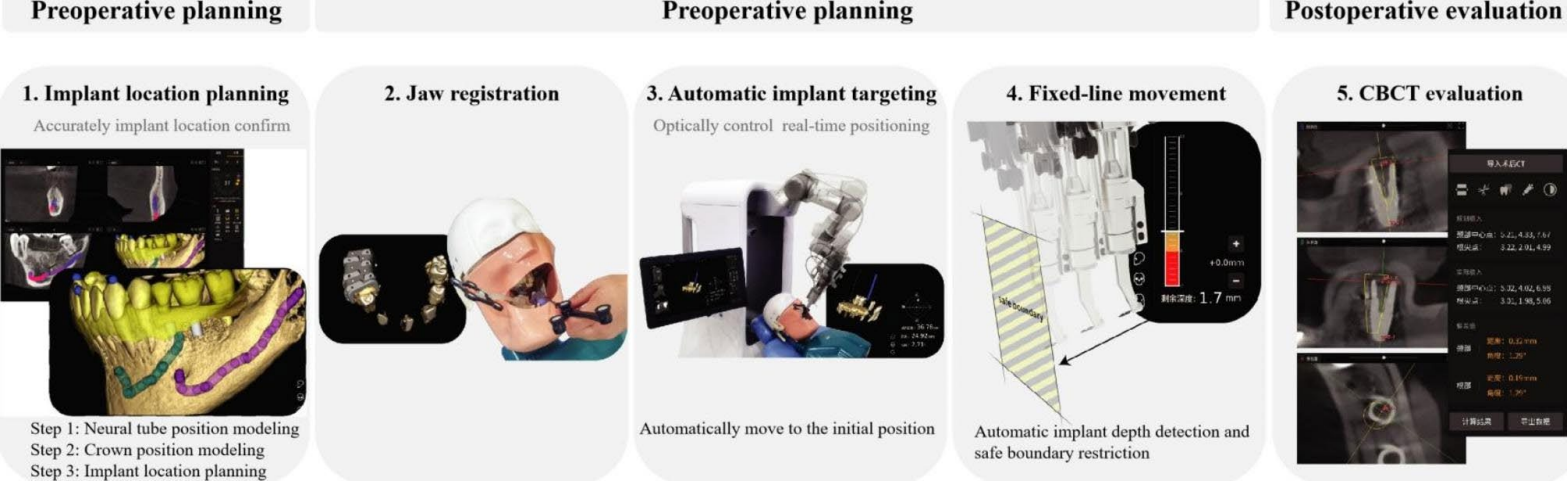
Experimental operation procedure with THETA dental implant robotic system

The THETA robotic dental implant system (Hangzhou Jianjia Robot Company, Hangzhou, China) is an integrated implant surgical robot and could complete dental implant surgery, which is composed of hardware including a mechanical arm, a binocular camera, an industrial control computer, an integrating platform, and an operation tool. The handpiece can be attached to the manipulator (Fig. 2).

A Jianjia U-shape silicone tube (Hangzhou Jianjia Robot Company, Hangzhou, China) was mounted on the edentulous area of the model with silicone impression material (DMG, Hamburg, Germany). The models were scanned with cone-beam computed tomography (CBCT) (Planmeca ProMax, Planmeca Oy, Helsinki, Finland), all scans were performed at 80 kV and 6.0 mA for 15 s (voxel size: 0.15 mm; grayscale: 15 bits; focal spot: 0.5 mm; and field of view: 12 × 9 cm), then imported into surgical planning software (Cycad DHC-DI, version: V2, Hangzhou Jianjia Robot Company, Hangzhou, China). Implants (NobelParallel CC) were placed virtually in each model. The implant procedure was designed according to the planning software. The experimental operational procedure was shown in Fig. 1. Importing the designed preoperative planning files into the THETA robot navigation operation system, the clinician can adjust the position of the implant if needed. Then enter the preparation of THETA equipment, confirm the placement of the equipment, adjust the position of the trolley and keep it fixed. Install the extremity array and the extremity implant handpiece at the end of the robotic arm to ensure the visibility of the required array during surgery. Enter the extremity calibration interface, install the drill calibration tool into the extremity implant handpiece, and use the calibration probe tip to select points on the drill calibration tool, two lines with four extremity feature point collections will be displayed on the drill calibration tool. Four feature points need to be collected. After the collection of feature points was completed, the probe tip is placed at any point in the groove to verify and calculate the accuracy of the current robot arm extremity calibration. After verification, remove the bit calibration tooling. Enter the U-tube matching interface. Reset the U-tube on the experimental model, fix the oral fixture on the appropriate tooth position of the model with temporary dental material to ensure that the array signal and calibration probe of the oral fixture can be captured and displayed by the binocular camera. Nine registration points on the surface of the U-tube were picked sequentially using the probe. After the registration points collection was completed, enter the accuracy verification interface. According to the prompt, use the probe to pick the verification points of the U-tube. If the acquisition errors of multiple verification points are less than 0.4 mm, the verification is qualified. Remove the U-tube after the accuracy verification. Enter the implant cavity preparation interface, select the appropriate implant drill, adjust the robotic arm to the appropriate posture, the operator holds the teaching button (the round button), and drags the robotic arm to the vicinity of the implant position (within a range of less than 10 mm), release the button, the end of the robotic arm is automatically positioned to the implant position, then presses the alignment button (the oval button) to perform the alignment movement in the planned axial direction of the implant. The operator can perform the pulling action in the axial direction to facilitate the cooling of the drill bit and the discharge of debris. During the alignment movement, the depth control can display the current drilling depth in real-time. When the planned drilling depth is exceeded, the depth control scale line will reach the red area, and the system will activate the safety boundary function to prohibit the end of the robotic arm from continuing to move downward. After reaching the planned drilling depth preparation, the operator releases the alignment button, presses the teaching button, drags the robotic arm out of the mouth, replaces the reamer in sequence, completes the cavity preparation and implants the implant. Take postoperative CBCT. The architecture of the robotic system was shown in Fig. 2.

### Yizhimei dynamic navigation-assisted implant surgery procedure

The Yizhimei computer-assisted dynamic navigation system (DHC-D12, Digital-health Care Co., Ltd., Suzhou, China) consists of a cart (including host, display, infrared tracking and positioning device, dynamic navigation system software), registration device, fixture device, reference board, and implant handpiece with locator and accessories. In addition, medical data including image progression and cast model scanning, preoperative planning, head models, and software for planning, calibration, and control are necessary to form the entire simulation system for surgery.

**Fig. 3:**
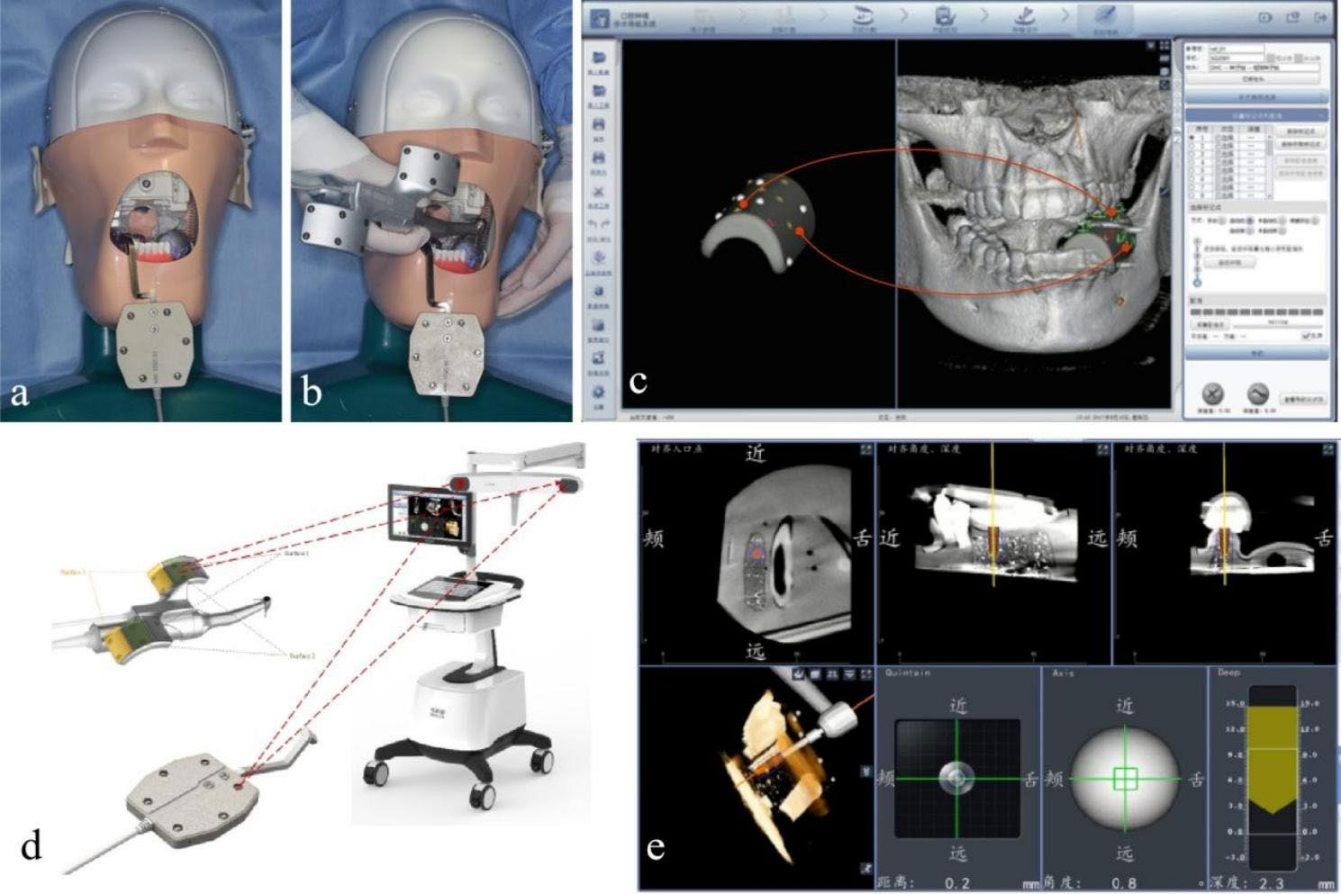
Model implantation using dynamic implant surgery. (a) U-tube mounted on maxilla with impression material; (b)The registration process: short drill clicks any 6 small ball pits on the registration device to complete the registration information collection; (c) U-tube registration; (d) Schematic diagram of dynamic navigation system; (e) Implant insertion assisted with dynamic implant surgery; the handpiece and mandibular were tracked with stereo infrared light camera; Precise operation under the control of implantation point, angle and depth

A U-tube (Digital-health Care Co., Ltd., Suzhou, China) was mounted on the edentulous model with silicone impression material. The models were scanned with CBCT and imported into surgical planning software (Yizhimei, Digital-health Care Co., Ltd., Suzhou, China). Enter the dynamic navigation implant system for preoperative planning, enter the real-time navigation interface, select the implant handpiece and reference plate, the reference plate is fixed on the adjacent tooth or the opposite side of the same jaw. Six zirconia pits on the U-tube were identified by the navigation implant mobile drill, the point-to-point registration was performed by calculating the distance between the position and the reference point, to identify and locate the surgical area. Remove the U-tube after registration. According to the guidance of the dynamic navigation system, the planting cavity was prepared and the implants were implanted. During the operation, the surgical operator can selectively observe the surgical approach and various parameters of the surgical area from all directions in time. According to the instructions of the software, the implant site, angle and depth can be dynamically adjusted to ensure that the implant results conform to the design plan. Postoperative CBCT was performed. The architecture of the dynamic implant surgery was shown in Fig. 3.

### Accuracy analysis

**Fig. 4:**
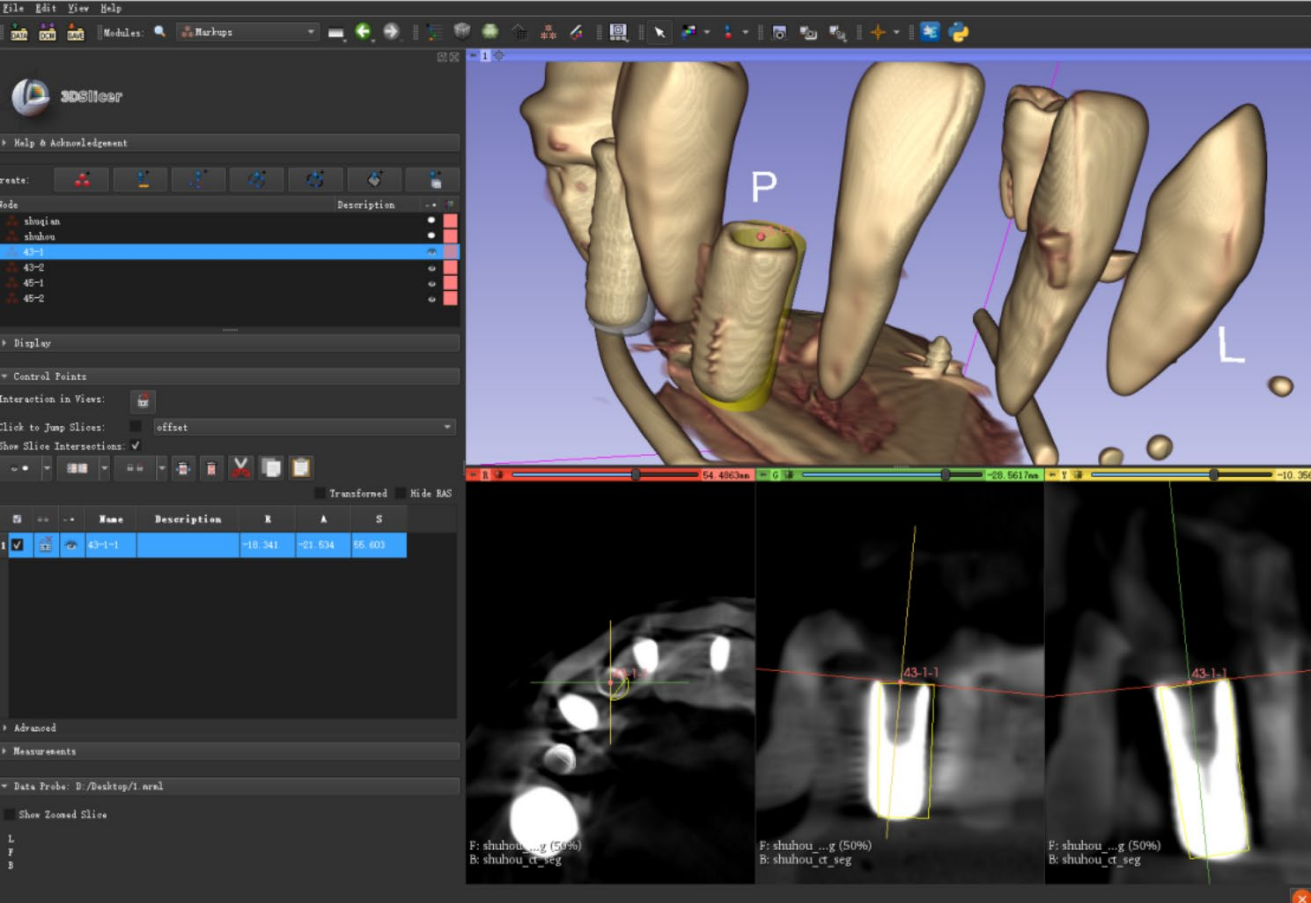
Fusion and calibration of preoperative and postoperative images by 3D Slicer

**Fig. 5:**
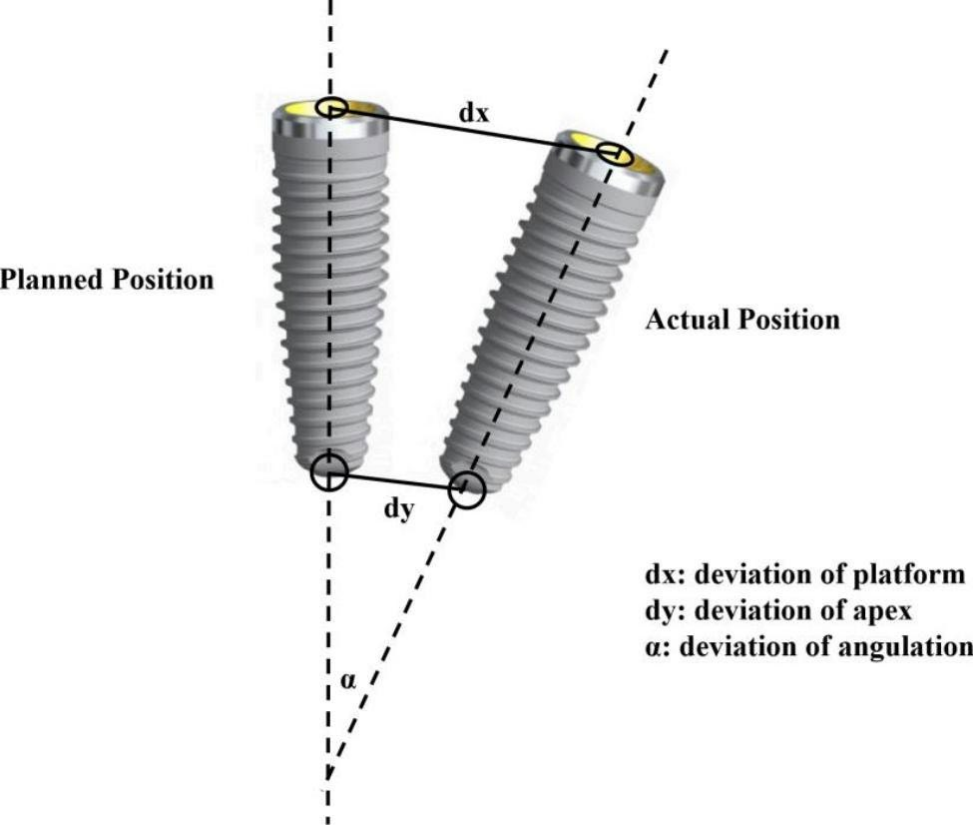
Illustration of deviations (platform, apex and angulation) of implants between pre-operation design and actual post-operation placement

After the implant was placed, postoperative CBCT was performed using the same parameters. The accuracy was analyzed by a open-source software 3D Slicer (Version 4.13, Harvard, Boston, USA, https://www.slicer.org) according to the method described by Talmazov G et al. [11]. The data of the preoperative implant design position was fused with the actual postoperative position image and a transformed alignment of more than three points was performed, which was checked by the best-fit algorithm alignment and adjusted if necessary (Fig. 4). The long axis of the planned and the placed implant positions were compared and measured for angular deviation as well as apical and platform distance (Fig. 5). The center of the implant platform and apex points of the designed and actual implant were marked and the straight line distance is recorded as dx and dy respectively. The angle between the long axis of the designed implant preoperation and the long axis of the actual implant postoperation was defined as the angular deviation (α). All measurements were conducted by one clinician who did not participate in the phantom surgery. To assess the intra-examiner reliability, duplicate registration was performed by the same examiners at an interval of 24 h. The mean value of measurements was used to calculate the deviation.

### Statistical analysis

The statistical analysis was conducted using SPSS 17.0 (IBM Corp, Armonk, NY, USA). All data were presented as mean, maximal/minimal value (max./min.), standardized deviation (SD) and 95% confidence interval (95% CI). The normality distribution of the data was evaluated using the Shapiro-Wilk test. The intra-group correlation coefficient (ICC) was used to evaluate the consistency of the same examiners measured at an interval of 24 h. The data conforming to normal distribution were expressed in the form of “x ± s”, and the differences were analyzed by independent sample t-test. The data that did not conform to normal distribution were analyzed using Mann-Whitney *U* test. A significant difference was defined as *p* < 0.05.

## Results

**Fig. 6:**
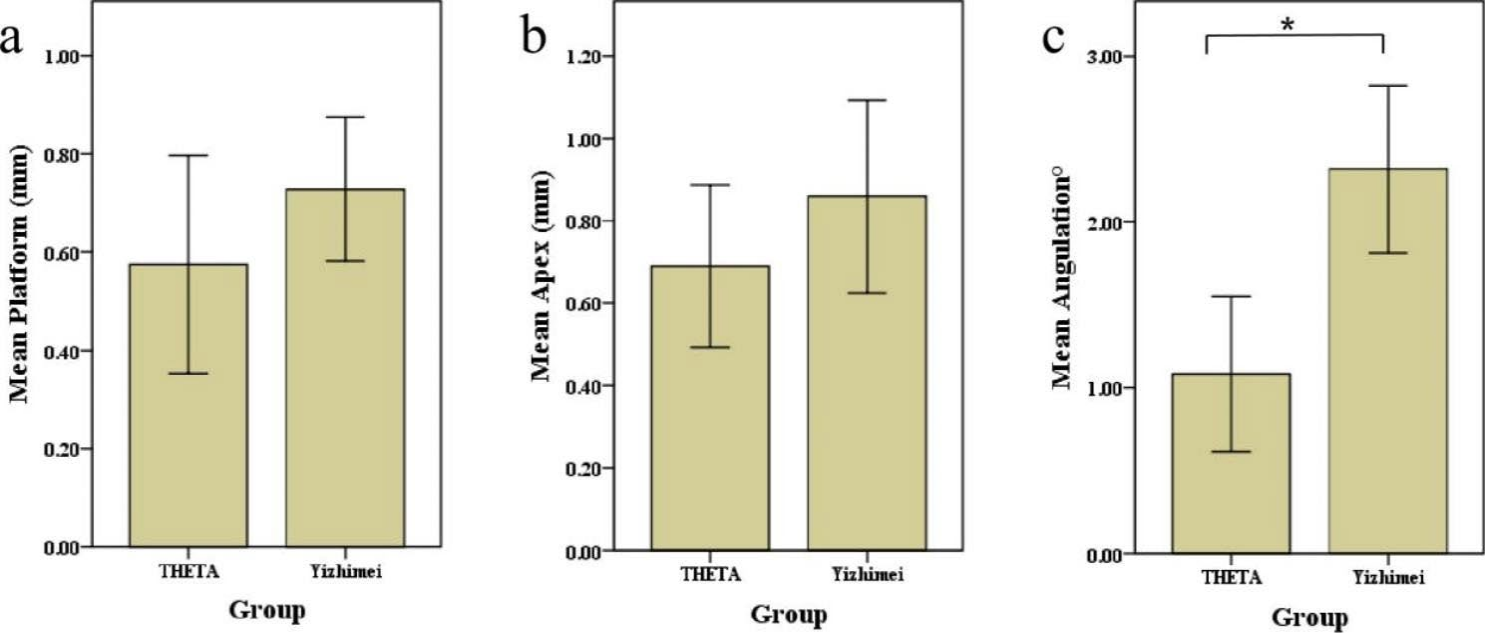
Implant deviations between THETA robotic system and Yizhimei dynamic system. Deviations of platform (a), apex (b) and angulation (c)

In total, ten implants were placed on the partially edentulous model using the THETA robotic system and the Yizhimei dynamic navigation system, respectively. The deviations in the platform and apex points and angle of the two groups were summarized in Tables 2 and 3. In Fig. 6, the mean deviations at platform, apex, and angulation of the two groups were compared. In the THETA group, the mean deviations between planned and postoperative implant position were 0.58 ± 0.31 mm at platform, 0.69 ± 0.28 mm at apex, and 1.08 ± 0.66° for angulation (Table 2). In the Yizhimei dynamic system group, there were also deviations in the planned and postoperative implant positions. The mean deviation was 0.73 ± 0.20 mm at platform, 0.86 ± 0.33 mm at apex, 2.32 ± 0.71° for angulation (Table 3). In terms of angulation deviation, implant placement with the THETA robotic system was significantly more accurate than Yizhimei dynamic navigation system (t = -6.252, *p* < 0.001) (Fig. 6). There was no statistical difference between the deviation in two groups at deviation of platform (t = -1.240, *p* > 0.05) and apex (t = -1.277, *p* > 0.05) (Fig. 6).

**Table 2:** Implant deviations using THETA robotic system

Deviation	Max	Min	Mean	SD	95%CI	
					Lower	Upper
Platform (mm)	0.98	0.11	0.58	0.31	0.35	0.79
Apex (mm)	0.99	0.23	0.69	0.28	0.49	0.88
Angulation°	1.96	0.24	1.08	0.66	0.61	1.55

**Table 3:** Implant deviations using Yizhimei dynamic navigation system

Deviation	Max	Min	Mean	SD	95%CI	
					Lower	Upper
Platform (mm)	1.12	0.49	0.73	0.20	0.58	0.87
Apex (mm)	1.35	0.40	0.86	0.33	0.62	1.09
Angulation°	3.74	1.66	2.32	0.71	1.81	2.82

## Discussion

The long-term success and survival of dental implants require precise positioning of the implant on the basis of the restoration. Computer-assisted systems of different technological approaches are currently used in clinical practice to improve the accuracy of implant placement, mainly including static surgical templateguided surgery, computer-assisted dynamic navigation, and robotic assisted dental surgical systems [12]. Static navigation techniques based on digital surgical guides could reduce the technical sensitivity of implant surgery, but have some limitations, such as long manufacturing time, high processing costs, and the inability to change the design during surgery. Moreover, in the narrow space of the oral cavity, digital surgical guides might affect the performance of dental implant surgery operations [4, 13]. The development of dynamic real-time navigation implant technology has broadened the scope of application of precision implants [14]. Computer-assisted dynamic navigation system allows monitoring the three-dimensional position of the drill and the location of important anatomical structures during implant surgery, which is particularly suitable for implant surgeries with complex local anatomical conditions and deeper implant sites without affecting the cooling effect during the implantation process [15, 16]. However, the dentist needs to monitor the real-time position of the drill or implant displayed on the screen, which is not intuitive [17, 18]. In addition, the mobile locator of the real-time dynamic navigator occupies a certain space, and its weight and volume are larger than the traditional free-hand implanted devices, which will affect the stability of the operation and increase the fatigue of the operator.

Dental implant surgery robots have evolved rapidly in recent years, integrating a computerized surgical planning platform, a visual surgical tracking platform and a robotic operating platform [19]. For dental robotic systems, the mechanical arm can automatically control the implant head and complete the preparation of the implant cavity according to the preoperative surgical planning, guided by navigation. The robotic system automatically prepares the hole according to the preoperative three-dimensional position planning and stops drilling automatically after the hole reaches the specified depth. The robotic arm can precisely move instruments in three-dimensional space, operate in a narrow oral cavity. Due to the introduction of the robotic arm, the implantation robot can avoid human error caused by operation fatigue, visual blind spot, and poor posture during artificial implant cavity preparation, which can further improve surgical precision and reduce the surgical complications [20, 21].

Previous studies have shown comparing with conventional implant surgery, static and dynamic navigation systems both have higher surgical precision. Block et al. [3] reported the deviation of the actual and designed position of the implants placed in vivo using the computerized dynamic navigation system. The mean deviation value of the angle was 7.69 ± 4.92°, the depth deviation of apex was 0.92 ± 0.55 mm, and the horizontal deviation of the apex was 2.21 ± 0.99 mm. Wagner et al. [22] reported that with surgical computer-aided navigation system, the deviation of platform, apex and angle of implants in vitro was 0.58 mm, 0.79 mm and 3.55°, respectively. Tencati et al. [ 23] investigated that the accuracy of static navigation surgery assisted by a digital surgical guide template was better than the freehand implant surgery. The results showed that in the group of static navigation surgery, the error value at platform was 1.16 ± 0.52 mm, the apex was 1.32 ± 0.61 mm, and the angle was 2.84 ± 1.60°. In contrast, the error value at platform, apex, and angle of implant placement was 1.86 ± 0.77 mm, 2.40 ± 1.00 mm, and 6.68 ± 4.06° respectively after the freehand implant surgery.

In this study, implant accuracy was evaluated using dynamic navigation surgery and a robotic system for implant surgery on a partially missing tooth model, and both systems showed excellent accuracy. The mean deviations between planned and postoperative implant position with THETA robotic system were 0.58 mm at platform, 0.69 mm at apex and 1.08° of angulation. In the group of Yizhimei dynamic navigation system, the deviations at platform, apex, and angulation were 0.73 mm, 0.86 mm and 2.32°, respectively. The high accuracy of our study was not difficult to predict since it was an in vitro study. The placement of the implants in the model does not involve the real oral environment, changes in the position of the patient’s head or tongue movements. In addition, the repetition of the operation under the same conditions reduces the operator’s random manipulation error.

In a dynamic navigation system, the drill and implant were controlled by the surgeon’s hand without any mechanical guidance instruments [24]. The implant accuracy relies on the eye-hand coordination skills and the interpretation of the data on the navigation monitor. In the clinical practice of dynamic navigation systems, operator experience becomes an important variable in determining accuracy [17, 18]. In this study, the implants were inserted in the same sites by a same dentist. Therefore, the operator’s error was largely minimized by the experience, which offered the implant surgery robot system advantage in accuracy assessment over the dynamic navigation system specified in this study. The implant surgery robot system has a mechanical arm with force feedback, which works in real-time human-robot collaboration during the operation. The operator can move the end of the robotic arm by hand, and the system can visualize the signals based on the tracking device to suggest the correct position of the device to the operator. The robotic system guides the operator to move the implanted handpiece to the correct site for hole preparation and implantation, which can avoid operational fatigue and visual blindness during manual hole preparation.

The length of the implant may significantly impact the precision of implant placement, particularly at the implant platform and apex. A template-guided implant placement study revealed that implant insertion with a length of 8 to 9 mm resulted in significantly higher precision than insertion with lengths of 10 to 11 mm and 12 to 13 mm, although angle deviation was unaffected by implant length [25]. Similarly, a recent research has shown that there is a statistically significant difference in the deviation of the apical and cervical regions during implant placement, depending on the implant diameter and length, when using digital surgical guides for dental implants [26]. In order to reduce the influence of this aspect, we utilized the same implant diameter and length for each paired group. In clinical practice, different implant sizes are selected based on the alveolar bone’s size. Therefore, we selected three commonly employed implant sizes of 8.5, 10, and 11.5 mm for the study.

The limitation of this study is referring to the relatively small sample size. Nicchio N. also utilized small sample sizes in their research [27]. However, to minimize errors, we conducted a plethora of preliminary experiments and exercises during the initial stages to ensure that the operator was highly experienced. We also performed handling and stability tests and calculated that 10 samples per group were sufficient. Furthermore, we employed the same model, CBCT scanning conditions, and bone density and implant size for each group in both experimental groups. All experiments were executed by the same operator.

The findings of this study have provided valuable insights into the comparative efficacy of the dental implant robotic system (THETA) and dynamic navigation system (Yizhimei) in clinical practice. Notably, the THETA system is distinguished by its use of a mechanical arm, while Yizhimei relies on a free-hand approach that demands a higher level of skill and experience from the practitioner. The study reveals that the angular deviation observed in the robotic system was superior to that of the dynamic navigation system, indicating that the fixed-line movement of THETA’s mechanical arm offers distinct advantages in dental implant surgery. These findings suggest that the THETA robotic system may hold significant promise as a tool for enhancing clinical outcomes in dental implantology, although additional research such as a prospective randomized study is necessary to further evaluate the accuracy of dental implant robots and their influencing factors. To date, dental robotics has made great progress, but it is still far from perfect yet. The intelligence of dental robotics is generally limited, and the operation is mainly assisted by doctors, with relatively simple functionality, the structure is usually complex and the volume is large, more widespread clinical application of this technology is expected in dentistry in the near future as the dental robotic systems hardware and software mature.

## Conclusion

Within the limitation of this in vitro study, the accuracy of implant placed using the novel implant dental implant robotic system (THETA) was superior to that of the dynamic navigation system (Yizhimei). In the future, the effectiveness and safety of the implant surgery robot can be verified through animal experiments and preclinical experiments for eventual application in clinical dental implant surgery.

## Data Availability

All data are calculated by the software itself. The datasets used and/or analysed during the current study available from the corresponding author on reasonable request.
